# Multiphoton Multispectral Fluorescence Lifetime Tomography for the Evaluation of Basal Cell Carcinomas

**DOI:** 10.1371/journal.pone.0043460

**Published:** 2012-09-11

**Authors:** Rakesh Patalay, Clifford Talbot, Yuriy Alexandrov, Martin O. Lenz, Sunil Kumar, Sean Warren, Ian Munro, Mark A. A. Neil, Karsten König, Paul M. W. French, Anthony Chu, Gordon W. H. Stamp, Chris Dunsby

**Affiliations:** 1 Photonics Group, Department of Physics, Imperial College London, South Kensington Campus, London, United Kingdom; 2 Department of Dermatology, Imperial College Healthcare NHS Trust, London, United Kingdom; 3 Department of Medicine, Imperial College Healthcare NHS Trust, London, United Kingdom; 4 JenLab GmbH, Jena, Germany; 5 CRUK London Research Institute, London, United Kingdom; Tufts University, United States of America

## Abstract

We present the first detailed study using multispectral multiphoton fluorescence lifetime imaging to differentiate basal cell carcinoma cells (BCCs) from normal keratinocytes. Images were acquired from 19 freshly excised BCCs and 27 samples of normal skin (*in & ex vivo*). Features from fluorescence lifetime images were used to discriminate BCCs with a sensitivity/specificity of 79%/93% respectively. A mosaic of BCC fluorescence lifetime images covering >1 mm^2^ is also presented, demonstrating the potential for tumour margin delineation.

Using 10,462 manually segmented cells from the image data, we quantify the cellular morphology and spectroscopic differences between BCCs and normal skin for the first time. Statistically significant increases were found in the fluorescence lifetimes of cells from BCCs in all spectral channels, ranging from 19.9% (425–515 nm spectral emission) to 39.8% (620–655 nm emission). A discriminant analysis based diagnostic algorithm allowed the fraction of cells classified as malignant to be calculated for each patient. This yielded a receiver operator characteristic area under the curve for the detection of BCC of 0.83.

We have used both morphological and spectroscopic parameters to discriminate BCC from normal skin, and provide a comprehensive base for how this technique could be used for BCC assessment in clinical practice.

## Introduction

Basal cell carcinoma (BCC) is most common in caucasian populations and has a high prevalence in the western world [1], [2] with a rising incidence in all age groups [3], [4].

Initial assessment is currently made on clinical examination and uncertainty concerning the diagnosis can only be resolved by histology at present. The tumor must first be excised, fixed, processed, sectioned and stained before it can be reviewed. Biopsies can be both uncomfortable and cosmetically disfiguring to the patient and time-consuming and expensive to the clinician. The need for histological confirmation is also required in Mohs micrographic surgery to ensure clear margins and is often required in assessing clearance or recurrence following non-invasive topical therapy for malignancies. A non-invasive imaging modality capable of producing optically sectioned images *in situ* with high spatial resolution and correlation with histology is highly desirable. A number of label-free imaging modalities have been developed for dermatological applications [5] including high frequency ultrasound, optical coherence tomography, confocal laser scanning microscopy and multiphoton tomography (MPT).

MPT is an emerging optical imaging technique [6] that excites fluorescence from the sample through the simultaneous absorption of two or more photons of infrared light. This process requires a high intensity of excitation light and so is confined to the tightly focused excitation spot. Images are generated by raster-scanning the excitation spot across the specimen in two dimensions. MPT offers a spatial resolution similar to histopathology at high power magnification (<1 µm lateral, <2 µm axial) resolution [7] and is available for clinical use *in vivo*.

The skin contains naturally occurring fluorophores that can be imaged using MPT without the need for exogenous contrast agents. These include collagen, elastin, melanin, keratins, porphyrins, NAD(P)H and flavins. Fluorescence intensity imaging using MPT can be used to study skin morphology with subcellular resolution [8], [9], [10]. Further discrimination between fluorophores and thus tissues can be gained using the fluorescence emission spectrum [11], [12] and fluorescence lifetime imaging (FLIM) [13], [14], [15], [16], which measures the rate of decay of the fluorescence signal following a short pulse of excitation light.

The aim of this study is to evaluate multispectral FLIM MPT as a means to discriminate BCCs from normal skin. Fluorescence intensity and FLIM images were collected from normal skin (*in & ex vivo*) and freshly excised BCCs using four emission spectral channels. The potential of this technique to provide improvements in diagnostic accuracy was then explored using visual architectural criteria together with cellular morphology and spectroscopic parameters. We identify the cellular morphology and spectroscopic parameters providing the greatest diagnostic discrimination and combine these to allow new semi- and fully-automated diagnostic algorithms to be tested for the first time.

## Results

Table 1 summarizes the patients and data used in the analysis. 19 patients with normal skin were imaged *in vivo*, the remaining samples in the study were imaged *ex vivo*. Patients had skin phototypes I-IV with a mean of 2.5 and 2.2 for normal skin and BCCs respectively. All BCCs except 2 had nodular components and 8 were mixed subtypes including infiltrative, superficial and pigmented. Skin was imaged from the scalp, face, neck, chest, forearm and back for BCCs and face, forearm, back and lower leg for normal skin.

**Table 1:** Patient characteristics and median lifetimes for each spectral channel.

					Median τ_mean_ (ps) (Inter-quartile range)			
Diagnosis	Patients (M/F)	Mean Age (Range)	Images	ROIs/Cells	Blue	Green	Yellow	Red
**BCC**	19 (11/8)	64 (44–86)	110	4259	2419	2624	1908	1448
					(1759–2773)	(2412–2819)	(1582–2200)	(1240–2013)
**Normal Skin**	27 (15/12)	42 (17–80)	122	6203	1797	2189	1380	1036
					(1282–2175)	(1656–2709)	(888–1974)	(709–1428)
**% difference from Normal**					34.6	19.9	38.3	39.8

**Abbreviations** ROI-region of interest, τ_mean_ - mean fluorescence lifetime, ps - picoseconds.

**Figure 1:**
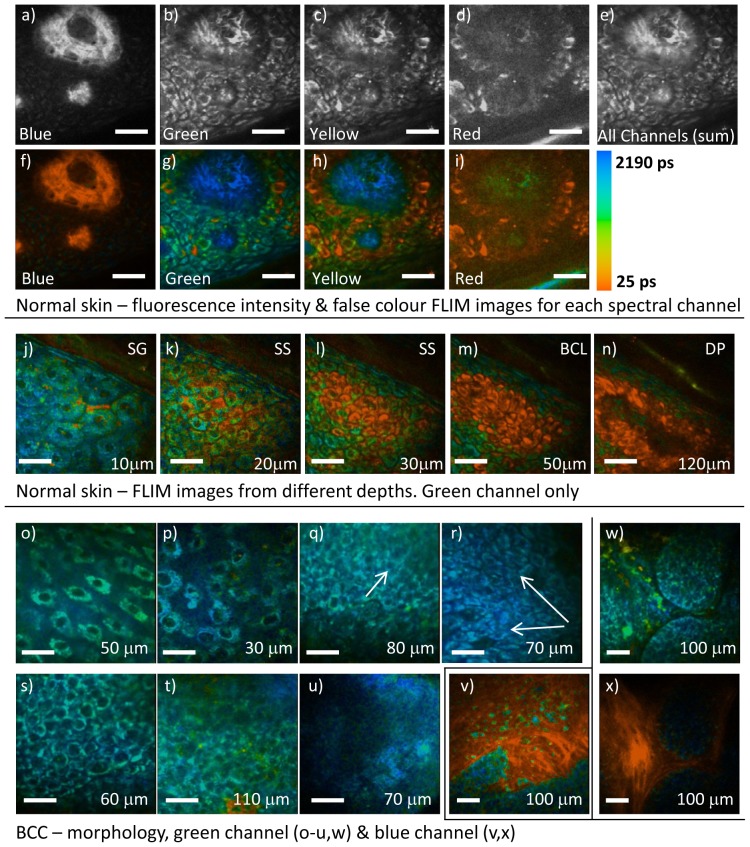
Multispectral fluorescence intensity and FLIM images acquired from normal skin and BCCs. (a–i) Fluorescence intensity and false color FLIM images from a single field of view acquired at a depth of 110 µm with all spectral channels taken near a dermal papilla from normal skin. (j–n) FLIM images taken from the green channel only of different depths within a sample of normal skin. (o–u) FLIM images taken from the green channel illustrating visual architectural features seen in BCC using MPT. (v) FLIM image taken from the blue channel of a BCC. (w,x) paired FLIM images taken from the green and blue channels respectively of a BCC nest. KEY SG-Stratum Granulosum, SS- Stratum Spinosum, BCL-Basal Cell Layer, DP-Dermal Papilla. Scale bar 25 µm.

Figure 1a–n demonstrates typical fluorescence intensity and FLIM images (encoding mean fluorescence lifetimes using a false color scale) taken from normal skin. Images in Figures 1a–i have been taken from the same field of view of normal skin using the 4 spectral channels, (see Materials and Methods, Instrumentation). Figures 1a–d show the images recorded in the 4 spectral channels revealing contrast that is not visible in the calculated total fluorescence intensity image (Figure 1e). The second harmonic generation (SHG) signal from collagen, seen in the tips of the dermal papilla, dominates the blue spectral channel (Figure 1a) and can be visually separated from the weaker intracellular fluorophores (c.f. Figure 1b–d). It is however difficult to visually separate the multiple fluorophores contributing to the green, yellow and red channels (Figure 1b–d) using the fluorescence intensity images alone. The addition of the fluorescence lifetime information (Figures 1f–i) allows NAD(P)H autofluorescence (blue-green on false color scale), dominant in the green spectral channel to be easily distinguished from melanin (short lifetime [17] red on false color scale), which is dominant in the red spectral channel. The yellow spectral channel includes fluorescence from flavins, NAD(P)H and melanin. The multiple FLIM spectral channels also allow SHG from collagen (with an instantaneous decay) seen in the blue channel and elastin (with a long fluorescence lifetime [18]), seen in the green/yellow channels to be distinguished from the same spatial location within the dermal papilla.

Figures 1j–n show FLIM images acquired in the green spectral channel from different depths within the same sample. Dendritic processes containing melanin can be identified in Figure 1j,k between the keratinocytes in the s.granulosum and s.spinosum. Deeper in the skin, the nucleus to cytoplasm ratio increases and more cells contain intracellular melanin with a short fluorescence lifetime. The highest concentration of melanin can be seen in the cells of the basal cell layer surrounding the dermal papillae (Figure 1n). Corresponding images from all spectral channels can be found in Figure S1.

Figures 1o–x illustrate the visual architectural changes seen in BCCs using MPT. We observe features described previously [19], [20] including ‘detached cells with enlargement of intercellular spaces’ (Figure 1o), ‘cells with irregular contours’ and ‘random arrangement of cells’ (both Figure 1p), ‘aligned elongated cells’ (Figure 1q, white arrow), ‘double alignment of monomorphous cells’ (Figure 1r, white arrows), ‘palisading’ (Figure 1w) and ‘sheets of cells intermingled with fibres’ (Figure 1v). The paired images in Figure 1w,x are taken from the green and blue channels respectively and show the proximity of BCC nests to the surrounding collagen fibres (‘cell islands surrounded by fibres’).

In addition to these features, we repeatedly saw a pattern of monomorphous cells, often heterogeneous in size, with large nuclear/cytoplasmic ratios, poorly defined cell margins and appearing to overlap. This new feature of ‘merging cells’ can be seen in Figure 1q,s,t,u and in the further image sets found in Figure S2.

All FLIM images were assessed by a dermatologist (RP) for the presence of at least one of the MPT diagnostic features for BCC described above and ‘merging cells’. This yielded a sensitivity/specificity of 79%/93%.

**Figure 2:**
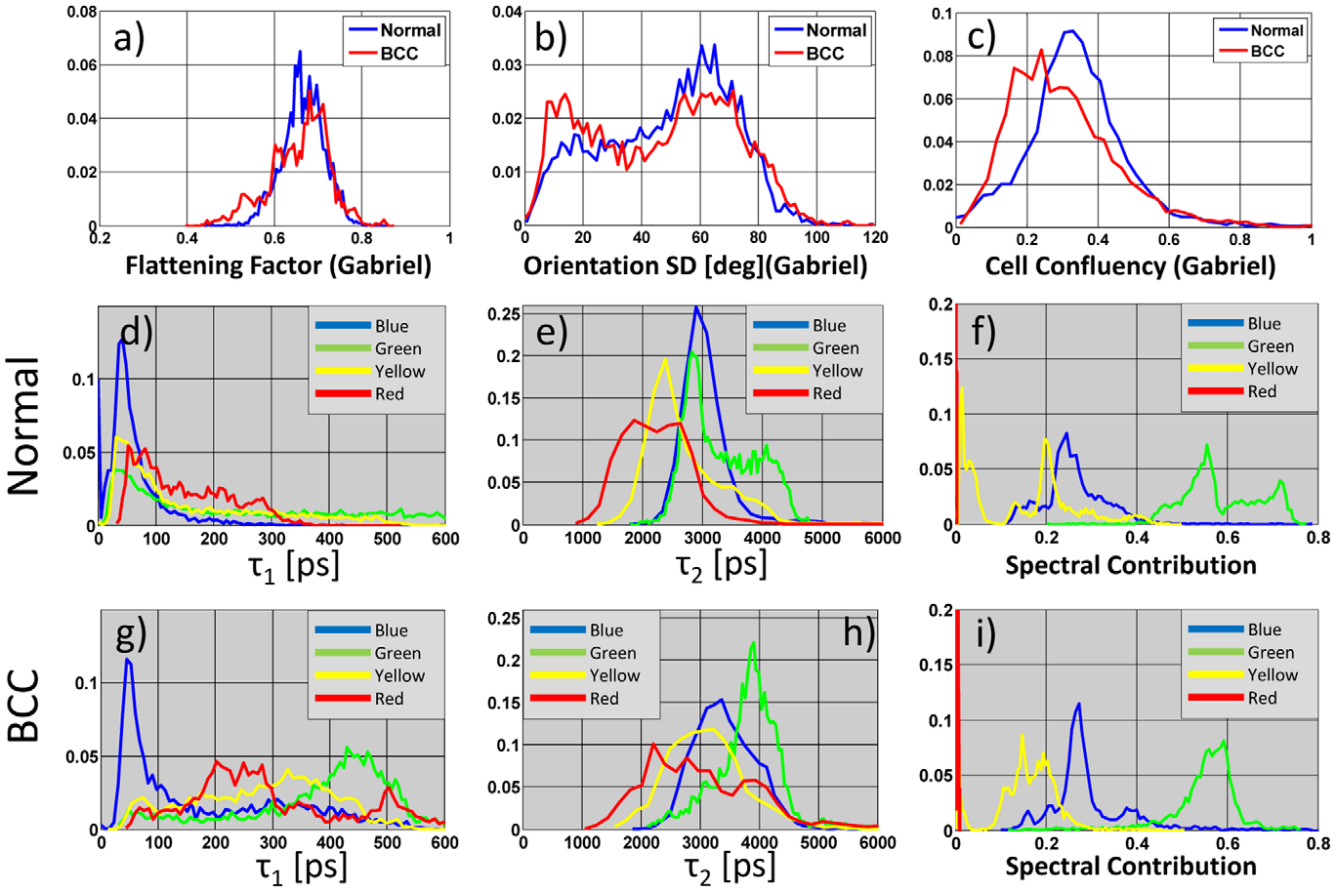
Histograms of morphology and fluorescence lifetime parameters for cells/ROIs in normal skin and BCCs. (a–c) Histograms of cellular morphology features demonstrating the difference between normal skin (coded Blue) and BCCs (Red) (d–i) Histograms of τ_1_, τ_2_, spectral contribution for all spectral channels for BCC and normal skin. Curves are color-coded according to channel “colors”.

A number of spectroscopic and cellular morphology parameters were calculated using manually defined regions of interest (ROI) corresponding to individual cells. The median ROI mean fluorescence lifetime is given for each spectral channel in Table 1, and the best discriminating parameters ranked by area under the curve (AUC) are listed in Table 2 together with the Cohen's d statistic. Histograms of several parameters are plotted in Figure 2a–f. A detailed description of all parameters, complete tables of all calculated values and their ranking by AUC are presented in Text S1, Table S1 and Table S2 respectively.

**Table 2:** The best discriminatory parameters ranked using the area under the curve (AUC).

Parameter	Index	Channel	Cohen's d	AUC
**Spectroscopic**	τ_1_	Red	1.44	0.82
	τ_1_	Blue	1.22	0.80
	τ_1_	Yellow	1.00	0.77
	τ_2_	Red	0.20	0.74
	τ_2_	Green	0.87	0.73
	τ_2_	Blue	0.38	0.73
	τ_1_	Green	0.83	0.72
**Cellular**	Cell confluency (Gabriel)	-	0.02	0.60
**morphology**	Orientation SD (Gabriel)	-	0.12	0.53
	Flatting Factor (Gabriel)	-	0.17	0.52

**Abbreviations** AUC – area under the curve, τ_1_ – short fluorescence lifetime decay component, τ_2_ – long lifetime component.

Figure 2a–c presents histograms of the three cellular morphology parameters, namely the flattening factor (ratio of minor to major axis length), orientation SD (the standard deviation of the ROI major axis angle of a cell and its neighbours), and the cell confluency (combined area of ROI and adjacent ROIs relative to intercellular space). The difference in the median value for the orientation SD and cell confluency between BCC and normal samples was found to be statistically significant at the 5% level using the Wilcoxon rank sum test (p = 0.03, 0.05 respectively), but was not significant for the flattening factor (p = 0.33). Both the AUC and Cohen's d statistics show that these distributions are not sufficiently separated for them to be used as the main components in multivariate discrimination. The difference in orientation SD reflects the increase in BCCs of aligned and elongated cells and of “palisade”-like structures. The cell confluency suggests that cells in BCCs are, on average, more sparsely arranged in an XY plane than those from normal skin. These differences are in accordance with visual assessments of tissue morphology discussed by Seidenari et al. [20] and above.

Histograms of spectroscopic and fluorescence lifetime parameters (Figure 2d–i) show a shift toward longer lifetimes for BCCs in τ_1_ (short) and τ_2_ (long fluorescence decay components) for all channels compared with normal skin. Strong peaks are seen in the BCC histograms at τ_1_ = 450 ps and τ_2_ = 4000 ps in the green channel, the peak in τ_2_ shifts from 2400 ps to 3000 ps in the yellow channel and new peaks in τ_1_ around 250 and 500 ps are present in the red channel in BCCs. The difference in median τ_mean_ between BCC and normal samples was found to be statistically significant to p<0.01 using the Wilcoxon rank sum test (p = 0.0013, 0.0049, 0.0061, 0.0006 in the blue, green, yellow and red channels respectively).

Analysis with respect to the fraction of the total fluorescence signal in each spectral channel (spectral contribution, Figure 2f) indicates a population of cells with a high contribution in the green channel in normal skin and a broader distribution in the yellow channel in normal compared to BCC. There is also an increase in spectral contribution in the red channel for BCCs, see also Table S1.

In order to develop a diagnostic algorithm using all of the spectroscopic and cellular morphology parameters, we employed principal component analysis based dimensionality reduction. Linear discriminant analysis was then applied to the first 4 principal components of the data, which allowed each ROI to be classified as either ‘Normal’ or ‘BCC’ using a leave one out approach, see Text S1. The fraction of cells/ROIs classified as ‘BCC’ was then determined for all patients. The separation of these variables' distributions (Figure S3a), as measured using the AUC, was 0.83. For example, if the threshold for classifying a patient as having BCC is set at having ≥30% of cells classified as BCC, then this yields a sensitivity/specificity of 89%/73%.

**Figure 3:**
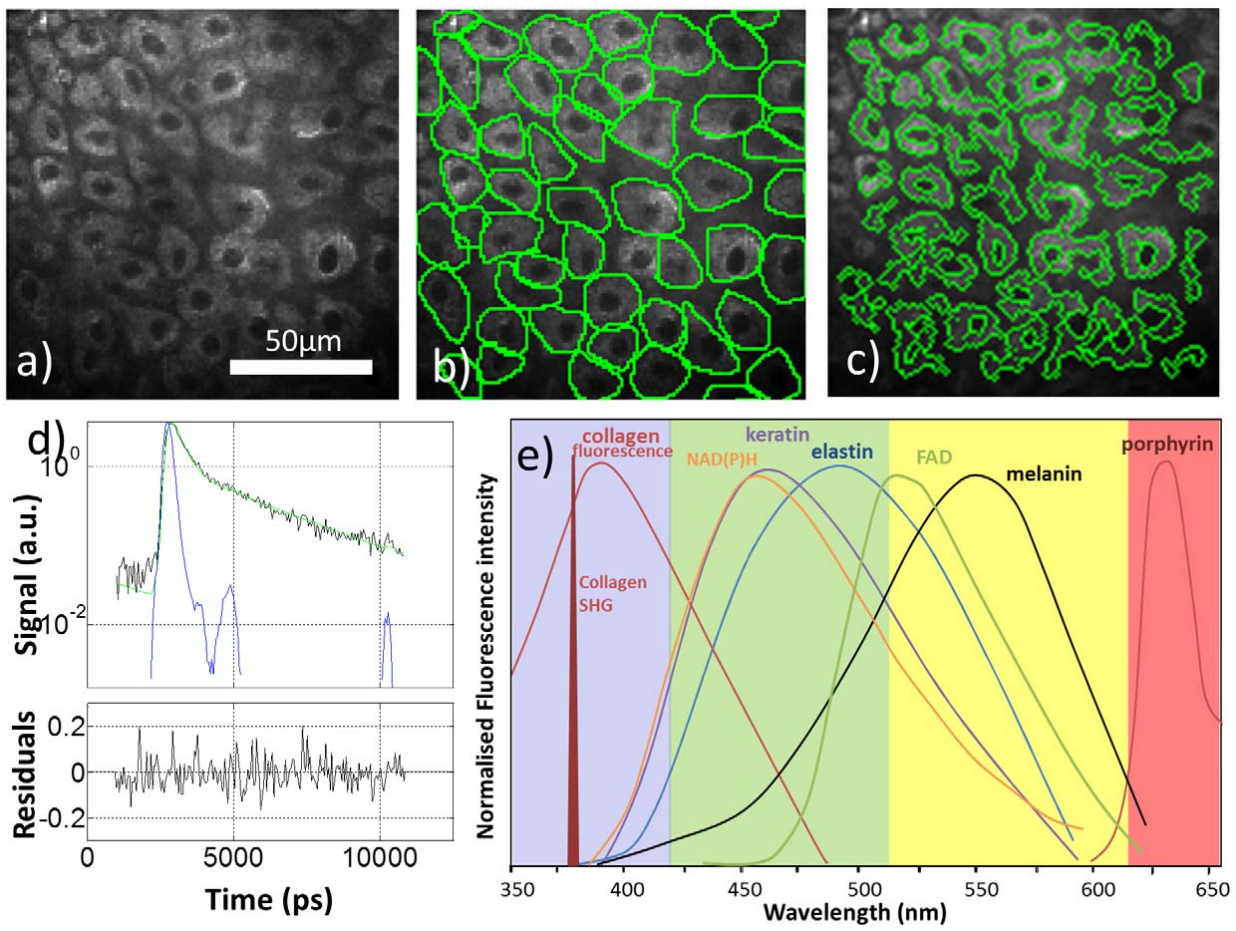
Exemplar segmented fluorescence intensity images, fitted fluorescence decay and fluorophore emission spectra. (a) Total fluorescence intensity image, same image with (b) manually and (c) automatically defined cellular regions of interest overlayed. (d) Top – exemplar fluorescence decay from one region of interest (black), biexponential fit to data (green) and instrument response function (blue). (d) Bottom – normalized residuals. (e) The emission spectra from endogenous fluorophores plotted in relation to the four spectral detection channels.

To further automate the discrimination we then replaced the step of manual segmentation with automatic ROI segmentation by the method of size-tuned non-linear top-hat detection [21]. This procedure identified 2343 BCC and 4034 normal ROIs. Here, the cellular morphology parameters were not included in the principal component analysis because the automatic ROI detection did not provide a clean outline of the cells (see figure 3(d)) and did not always correctly identify individual cells. However, the cellular morphology parameters only provide very weak discrimination between normal and BCC, see table 2, and so their exclusion is not expected to significantly affect the outcome. In fact, although the resulting fraction of cells/ROI's classified by this approach as ‘BCC’ (Figure S3b) is slightly different to that obtained with manual segmentation, the separation of these two distributions also yielded an AUC of 0.83. One might expect the discrimination based on automatically segmented ROIs to score lower than the one based on manual segmentation, due to the loss of information on the cellular morphology, the occasional grouping of more than one cell into the same ROI and erroneous inclusion of some extracellular fluorescence by the segmentation algorithm. In practice, however, we do not expect the exact shape of the ROI around a single cell to greatly affect the spatially integrated fluorescence decay from that ROI or the fluorescence lifetime parameters derived from it because the fluorescence decay profile is reasonably uniform across individual cells, see e.g. figure 1, and the erroneous inclusion of extracellular fluorescence makes only a small contribution to the total signal analysed. We note that, although the automatic segmentation is not perfect, it does allow bi-exponential fluorescence decay parameters to be measured at multiple regions across an image and is much less time-consuming than manual segmentation, which is important if this method is to be employed in a clinical setting. Further work and larger numbers of patients/samples will be required to compare the relative performance of these two methods more precisely. An analysis that did not include any image segmentation (see Text S1) also resulted in a reasonable sensitivity/specificity of 72%/84% respectively.

**Figure 4:**
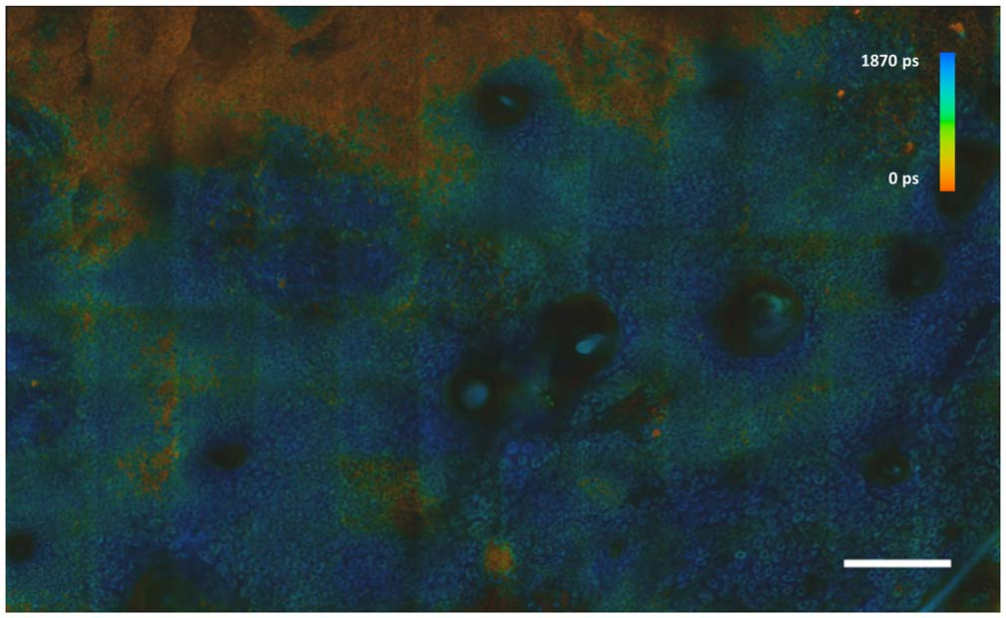
False color FLIM image from the green channel of a BCC consisting of 12×8 fields of view covering an area of 1.86×1.24 mm^2^. Bar 0.2 mm.

To observe the variation of spectroscopic and morphological parameters of a lesion across a larger field of view than is possible with our MPT instrument (350 µm×350 µm^2^), which is designed for use at high magnification, we employed a motorized stage to move *ex vivo* samples within the x,y plane and assembled mosaics of high resolution images. Figure 4 shows an example comprising 12×8 individual FLIM images from the green spectral channel acquired from a BCC, illustrating the potential of this technique to be used for high resolution label-free histology over a large field of view.

## Discussion

We have shown that morphological features described by Seidenari et al. [20] together with the newly proposed feature of ‘merging cells’ provide a good specificity and sensitivity for identifying BCCs from FLIM images using a visual architectural analysis. The multispectral FLIM images aided the identification of many features, especially when assessing cells within fibres, see e.g. Figure 1v–x.

In our cell-based spectroscopic analysis, we observe longer mean fluorescence lifetimes in all spectral channels for BCC compared to normal in agreement with earlier non-spectrally resolved FLIM MPT studies of BCC [16], [22], [23]. It is important to note that since normal keratinocytes are present within BCC, many cells/ROIs in the BCC FLIM images will have actually been normal cells, yet statistically significant differences were observed between groups in spite of this. From a spectroscopic perspective, it could be argued that the observed fluorescence lifetime contrast is due to a higher concentration of melanin (with a short fluorescence lifetime) in normal samples. We do not believe this to be the case because our BCC dataset includes pigmented BCCs and we observe well matched spectral contributions between groups in the melanin dominated yellow and red spectral channels (see Table S1). It is interesting to note that the spectroscopic parameters providing the highest discrimination (AUC) between groups are the fluorescence lifetimes of the red and yellow channels. We speculate that this could be melanin changing its fluorescence lifetime or due to the presence of an additional fluorophore such as porphyrins in BCCs. Further research is required to elucidate the origin of this contrast and we believe that multispectral MPT FLIM provides a useful tool to address this.

Previous *in vivo* measurements in a hamster cheek pouch model of oral cancer by Skala et al. [24] show a decrease in the fluorescence lifetime of NAD(P)H and flavins associated with malignancy. Our measurements show the opposite trend, which may be due to differences between animal model and human, in disease pathophysiology or the absence of melanin in mucosal epithelium.

Potential confounding factors in fluorescence lifetime measurements include [13] imaging depth in the skin (*τ*_mean_ decreases with depth) and patient age (*τ*_mean_ increases with age). In our study, image depths were well matched between groups (62 v. 65 µm mean cell/ROI depth) and are not expected to influence the results significantly. In a previous study of two very distinct age populations (20–35 and >60 years) using a single spectrally broad FLIM channel [13], mean fluorescence lifetimes were observed to differ between age groups by 109 ps for upper skin layers and by 210 ps for lower layers. In our study the age disparity is much lower (mean age 42 v. 64, Table 1) and the observed changes in fluorescence lifetime between normal and BCC are much larger (394–584 ps, Table 1).

In order to verify that it is reasonable to combine data from normal skin acquired *in vivo* with that acquired *ex vivo*, we studied how the fluorescence lifetime changes with time following surgical excision. The only previous such study on human skin was performed by Sanchez et al. [15] and this paper provides a detailed discussion of the many factors that can affect the fluorescence lifetime. Sanchez et al. observed skin over a timescale of 7 days and showed that the fluorescence lifetime increased over this period. Therefore, in order to study these changes over the timescale of hours relevant for our study, we repeatedly imaged samples of freshly excised normal skin at the same field of view over a three hour period. The mean fluorescence lifetime averaged over four samples decreased from the initial value by 7.2%, 1.6%, 2.9% and 8.6% in the blue, green, yellow and red spectral channels respectively. These changes are in the opposite direction to the observations by Sanchez et al. (which were carried out over days rather than hours) and, importantly, are all much smaller than the differences seen between diagnostic groups (34.6, 19.9, 38.3 and 39.8% respectively, Table 1).

As a second step to verify that it is reasonable to combine normal skin imaged *ex vivo* and *in vivo*, we directly compared the median ROI/cell mean lifetime between these two groups and the *ex vivo* samples were found to have a longer fluorescence lifetime by 5.9%, 7.9%, 10% and 13% in the blue, green, yellow and red spectral channels respectively. However, these changes are again smaller than the differences seen between BCC and normal skin (see Table 1). When we consider the median mean fluorescence lifetime per sample, see Figure S5, the difference between *ex vivo* and *in vivo* measurements was found to be not statistically significant using the Wilcoxon rank sum test (p = 0.98, 0.70, 0.63 and 0.23 in the blue, green, yellow and red spectral channels respectively). As reported above, the difference in median mean lifetime between the BCC samples and the combined *in vivo* and *ex vivo* normals was found to be statistically significant using the same test (p<0.01 in all spectral channels).

Combining the subcellular resolution of multispectral MPT FLIM with a motorized stage to facilitate imaging over large fields of view provides a means to identify small nests of BCCs in normal skin, such as those seen with infiltrative BCCs, whose margins are notoriously difficult to define. Currently the manual acquisition and processing of montage data is slow with data acquisition, FLIM data analysis and montaging requiring approximately 100, 4 and 60 minutes respectively for 12×8 images. In the future, full automation of the image acquisition process would reduce the image acquisition time to ∼45 minutes. Further reductions in image acquisition time may be possible by reducing the time required to acquire each sub-image, at the expense of signal-to-noise ratio in the final image. A wide range of sophisticated image stitching algorithms exist that could be used to greatly speed up the montaging compared to the current manual image stitching. While the current depth of penetration of MPT is too limited to assess the margins of many infiltrative BCCs *in vivo*, its high sensitivity, specificity and high spatial resolution can be exploited by stitching multiple images together to provide an extended multidimensional mosaic of such lesions. This could be useful for clinical *ex vivo* margin assessment, e.g. during Mohs procedures.

This work demonstrates the diagnostic potential of multispectral MPT FLIM for the evaluation of BCC and its margins. We have assessed existing MPT features and, together with a newly proposed feature (‘merging cells’), demonstrated that visual assessment of the images provides a sensitivity/specificity of 79%/93%. In addition, we have used multispectral FLIM to perform the first detailed analysis of the discrimination afforded by a wide range of spectroscopic and cell-based morphologic parameters and ranked them by their discriminatory power (AUC). This information will be crucial to guide the development of more accurate, faster and lower cost imaging modalities.

We report the first semi- and fully-automated diagnostic algorithms based on manual and automatic segmentation of multispectral MPT FLIM images. Manual segmentation of cells followed by principal component analysis based dimensionality reduction and yielded an AUC of 0.83 for the discrimination of BCC from normal skin. In the future, the combination of automatically calculated spectroscopic information together with manually identified visual architectural features could lead to even higher diagnostic accuracies.

## Materials and Methods

### Ethics Statement

The study was approved by the Riverside NRES London ethics committee (Ref 09/H0706/28 & 83) and conformed to the Declaration of Helsinki protocols.

### Instrumentation

Multiphoton imaging was performed using a modified DermaInspect® (JenLab GmbH, Jena, Germany), a CE marked two photon laser scanning tomograph [7]. Fluorescence was collected by four H7422P-40 photomultiplier tubes (Hamamatsu Photonics K.K., Japan) and recorded using time correlated single photon counting into 256 time bins (SPC-830, Becker & Hickl, GmbH, Germany). The four spectral channels were defined as 360–425 nm (blue), 425–515 nm (green), 515–620 nm (yellow) and 620–640/655 nm (red), see Figure 3e. Further description of the system is included in Text S1 and Figure S4.

### Patients and Samples

Patients with suspected BCCs and volunteers attending clinics at the Department of Dermatology at Imperial College Healthcare NHS Trust were recruited. Patients gave written informed consent to participate. Patients with the following criteria were excluded: age 18 years or less, in state custody, carrying a blood borne infection, having a photosensitive skin disorder, or currently pregnant.

Freshly excised tissue samples were typically 5–10 mm in depth and always consisted of the whole epidermis and dermis, to the subcutaneous fat. Samples were rinsed with Hanks Balanced Salt Solution buffer without phenol red, calcium or magnesium (Gibco®, Invitrogen™, CA, USA), its surface moistened with the buffer solution and placed on damp gauze in an inverted glass bottomed petri dish (80–170 µm cover slip Matek®, MA,USA) immediately following excision.

Images of normal skin were taken from healthy patients *in vivo* and normal margins of *ex vivo* tumor samples. *Ex vivo* normal skin was only imaged from well-defined skin tumors that were excised with a wide surgical margin of normal skin. This normal skin was sufficiently distant from the tumor site to not be affected and appeared normal by clinical visual inspection of the surface. *Ex vivo* samples were kept at room temperature and imaged 66±38 mins (mean±s.d.) after excision.

The diagnoses of all excised samples were confirmed histologically.

### Imaging

The excitation wavelength was fixed at 760 nm and the power was adjusted according to the depth of imaging (<50 mW). Images from several depths and sometimes more than one field of view were taken from within each lesion. Each FLIM image was either 128×128 or 256×256 pixels and was acquired over 25.5 s. The instrument response function was recorded daily from gold nanorods [25] in all spectral channels simultaneously.

The cellular layers of the epidermis were targeted for imaging. Images were taken at ∼10 µm intervals until the dermis was reached or until the signal was too low to resolve a useful image. The mean depth imaged per sample was 65 µm for the BCCs and 62 µm for normal skin.

### Data Analysis

FLIM images were generated by fitting a single exponential decay to the fluorescence from each spectral channel of each pixel in the image using software written in Matlab® (R2010b, The Mathworks Inc.,USA).

Regions of interest (ROI) were defined manually in each image corresponding to each cell using software written in Labview 7.1 (see Figure 3b,c) [26]. The spectral contribution was calculated for all ROIs. The spatially integrated fluorescence decay for each ROI in each channel (e.g. Figure 3d) was fitted to a double exponential model using non-linear least squares minimization. ROI fluorescence decay curves from a single spectral channel containing fewer than 1000 photons were excluded from the analysis as they contained too few photons to reliably fit to a double exponential model. ROIs containing <1000 photons in any spectral channel were excluded from the discriminant analysis for the same reason. Two patients (one normal, one BCC) did not have any ROIs with >1000 photons in all spectral channels and so were excluded from the discriminant analysis. See Text S1 for further details.

Automatic image segmentation was performed using size-tuned non-linear top-hat detection that has been described previously [21]. Briefly, this method applies a pixel-wise transformation to the image (formula A2.1 in [21]) that enhances the brightness of a pixel if its close vicinity is also bright and its distant vicinity is dim. The transformed image is then thresholded at a user defined value. The resulting binary images are then morphologically smoothed using the MATLAB ‘imopen’ function and spatially distinct regions identified. These regions are then size-sieved using standard MATLAB image processing functions to remove objects smaller than a user-defined threshold. The same procedure, including all thresholding parameters, was applied uniformly across all images.

### Image montaging

A motorized microscope stage (Scan IM 120×80, Märzhäuser Wetzlar Gmbh, Germany) was used for the sequential acquisition of data from several fields of view from an *ex vivo* sample (without the use of the metallic coupling ring). A total of 12×8 fields were acquired. Each image in the montage was acquired over 25.5 s and using 256×256 pixels. The manual saving and initiation of stage translation required ∼30 s therefore the total acquisition time for the montage of 12×8 images was ∼100 minutes.

Fitting a single exponential decay model to every pixel in an image required approximately 2.5 s using an 8-core, 3.16 GHz PC with 32 GB RAM running MATLAB R2011a under Windows 7, 64 bit. Therefore the FLIM decay fitting process required a total of ∼4 minutes for the entire montage in one spectral channel.

Due to backlash when reversing the direction of stage motion, fully automatic image stitching using the programmed stage translation distance did not provide satisfactory results. Therefore, semi-manual image registration was employed using a software package developed in-house in LabVIEW, which required approximately 1 hour to align the whole montage.

### Statistics

Statistics were performed using SPSS 18 (SPSS Inc. IL, USA) and MATLAB®. See Text S1 for further details.

## Supporting Information

Figure S1 **Spectrally resolved FLIM images from a sample of normal skin taken at several depths.** (Figure 1 j–n shows only the 425–525 nm (green) channel of this data).
(PDF)

Figure S2 **ultispectral FLIM images taken at multiple depths from (a) a nodular/superficial BCC and (b) a nodular/pigmented BCC.**
(PDF)

Figure S3 **Histograms of the fraction of cells classified as BCC for the BCC and normal groups using both manual (a) and automatic (b) segmentation.**
(PDF)

Figure S4 **Schematic of instrument.** Key-DCSP - dichroic short pass, DCLP – dichroic long pass.
(PDF)

Figure S5 **Bar chart showing sample median ROI/cell mean fluorescence lifetime for BCCs,** ***in vivo*** **normal and** ***ex vivo*** **normal skin for the four spectral detection channels.** Error bars indicate value of 25^th^ and 75^th^ percentile.
(PDF)

Table S1 **Summary of all spectroscopic parameters calculated for each ROI/cell.**
(PDF)

Table S2 **The AUC for all calculated spectroscopic (a) and cellular morphology parameters (b).**
(PDF)

Text S1 **Supporting Materials and Methods.**
(PDF)
