# Supplementary material for: Multiphoton Multispectral Fluorescence Lifetime Tomography for the Evaluation of Basal Cell Carcinomas
Source: PLoS One. 2012 Sep 11;7(9):e43460. doi: 10.1371/journal.pone.0043460 (PMC3439453; doi:10.1371/journal.pone.0043460)

Figure S1 - Spectrally resolved FLIM images from a sample of normal skin taken at several depths. (Figure 1 j-n shows only the 425-525 nm (green) channel of this data.)

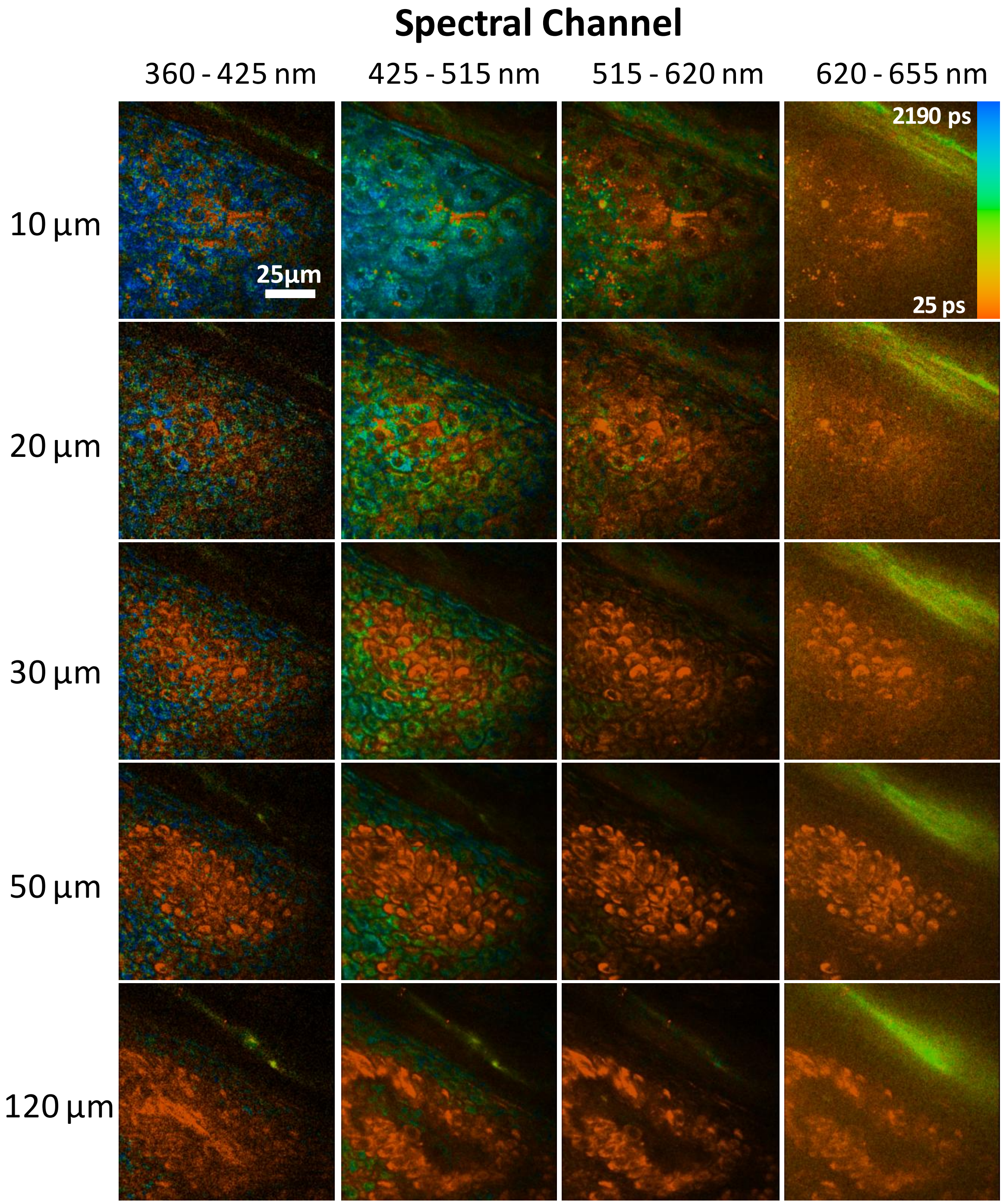

Supplement: Figure S1 — Spectrally resolved FLIM images from a sample of normal skin taken at several depths. (Figure 1 j–n shows only the 425–525 nm (green) channel of this data). (PDF) [file pone.0043460.s001.pdf]
