# Supplementary material for: Multiphoton Multispectral Fluorescence Lifetime Tomography for the Evaluation of Basal Cell Carcinomas
Source: PLoS One. 2012 Sep 11;7(9):e43460. doi: 10.1371/journal.pone.0043460 (PMC3439453; doi:10.1371/journal.pone.0043460)
Supplement: Figure S2 — ultispectral FLIM images taken at multiple depths from (a) a nodular/superficial BCC and (b) a nodular/pigmented BCC. (PDF) [file pone.0043460.s002.pdf]

Figure S2 - Multispectral FLIM images taken at multiple depths from (a) a nodular/superficial BCC and (b) a nodular/pigmented BCC.

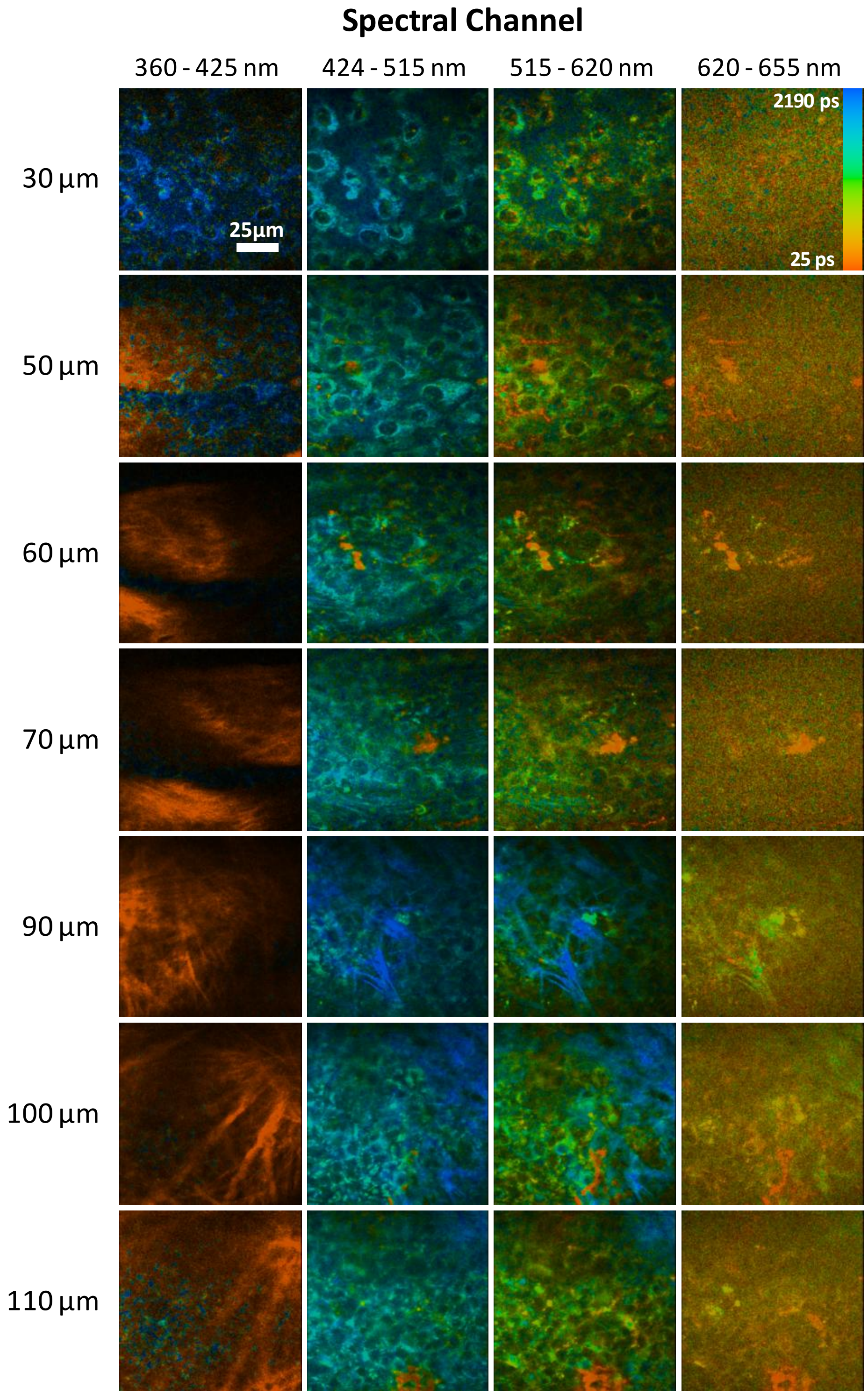

(a)

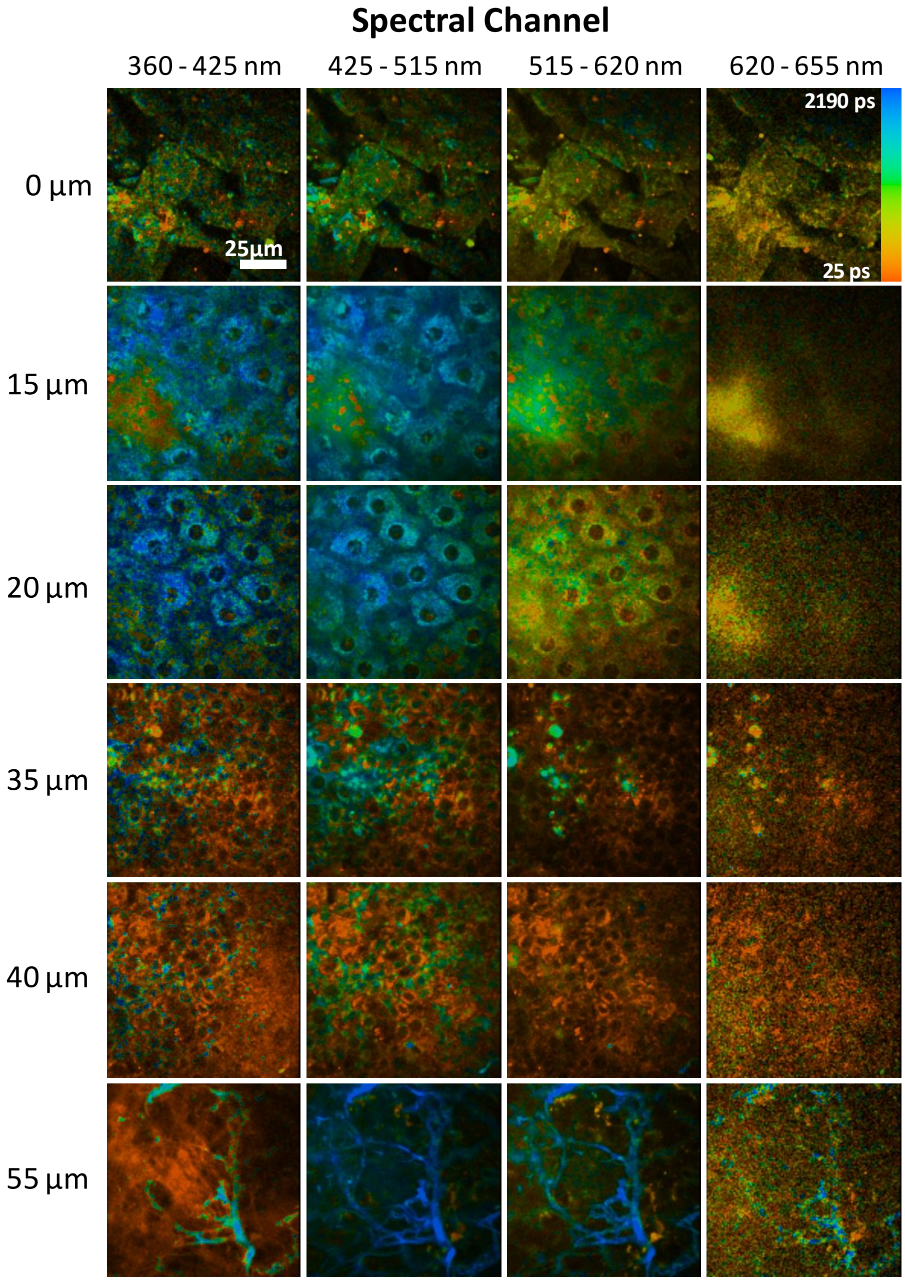
Spectral Channel
360 - 425 nm
425 - 515 nm
515 - 620 nm
620 - 655 nm
2190 ps
25 ps
0 µm
15 µm
20 µm
35 µm
40 µm
55 µm
25µm

(b)
