# Supplementary material for: Multiphoton Multispectral Fluorescence Lifetime Tomography for the Evaluation of Basal Cell Carcinomas
Source: PLoS One. 2012 Sep 11;7(9):e43460. doi: 10.1371/journal.pone.0043460 (PMC3439453; doi:10.1371/journal.pone.0043460)

Figure S3 - Histograms of the fraction of cells classified as BCC for the BCC and normal groups using both manual (a) and automatic (b) segmentation.

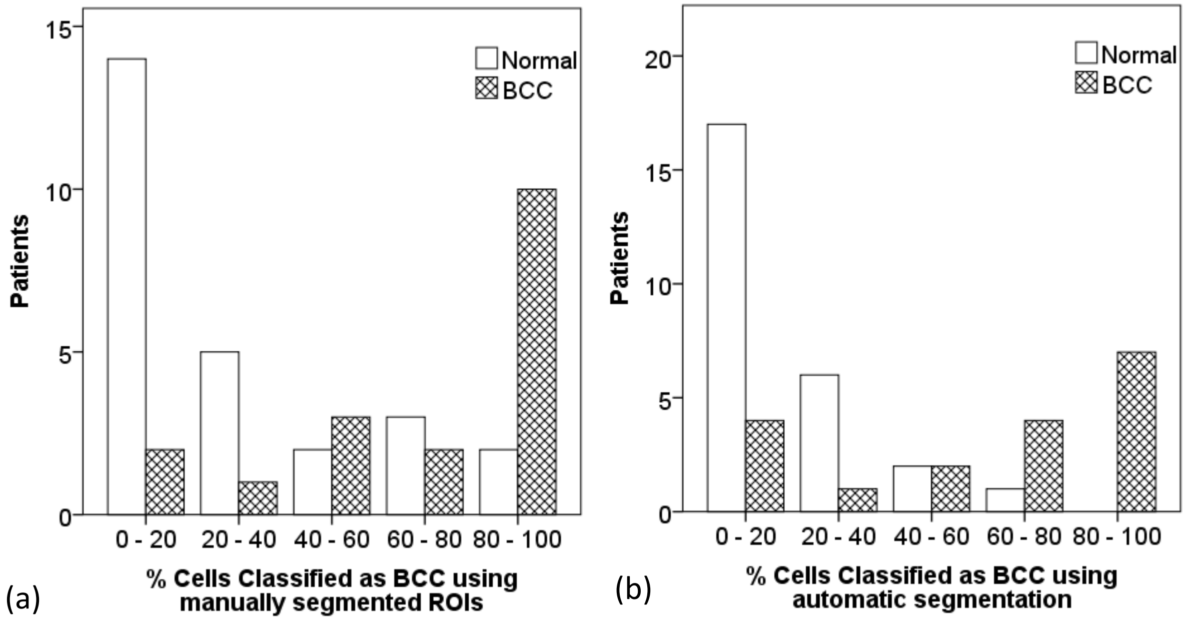

Supplement: Figure S3 — Histograms of the fraction of cells classified as BCC for the BCC and normal groups using both manual (a) and automatic (b) segmentation. (PDF) [file pone.0043460.s003.pdf]
