# Supplementary figures and images for: Multiphoton Multispectral Fluorescence Lifetime Tomography for the Evaluation of Basal Cell Carcinomas
Source: PLoS One. 2012 Sep 11;7(9):e43460. doi: 10.1371/journal.pone.0043460 (PMC3439453; doi:10.1371/journal.pone.0043460)

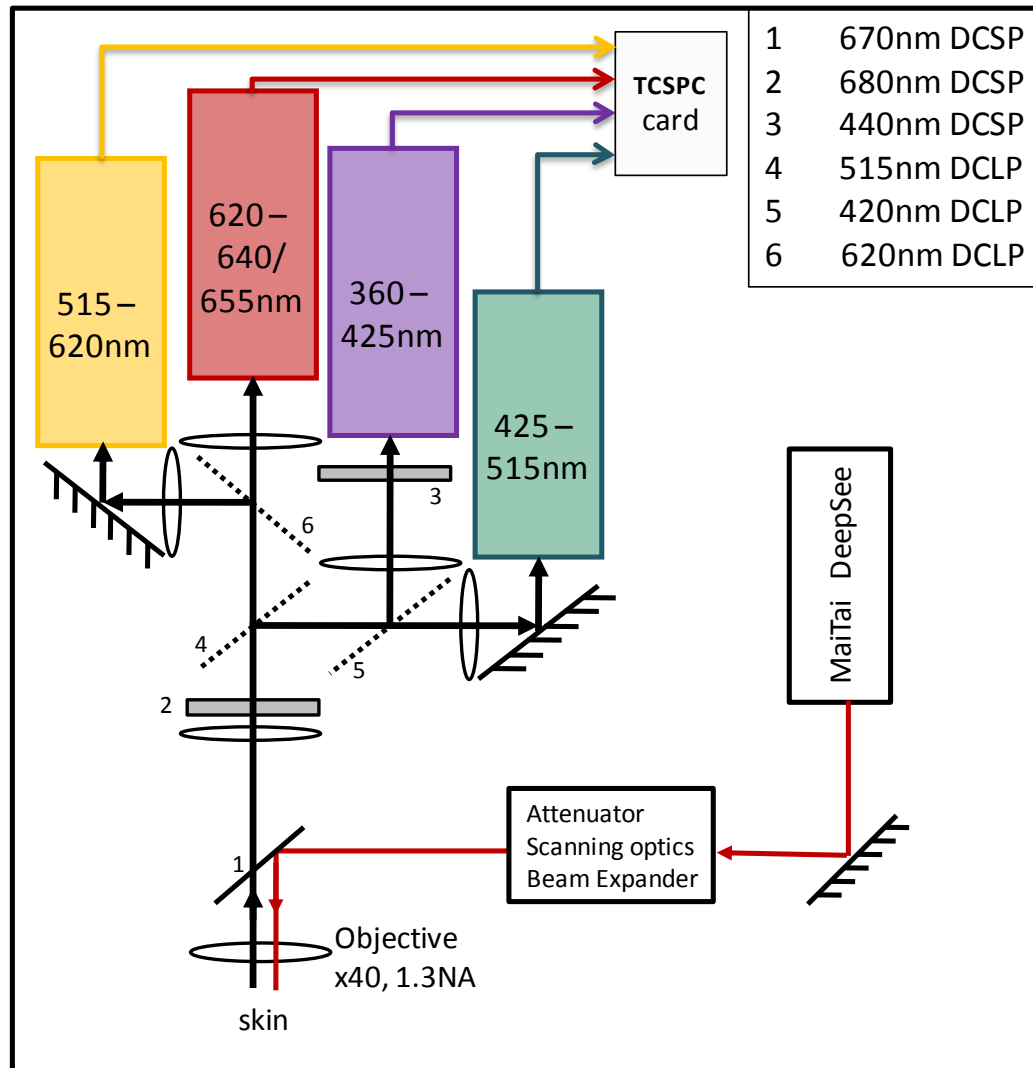

**Figure S4-** Schematic of instrument. Key-DCSP - dichroic short pass, DCLP – dichroic long pass

Supplement: Figure S4 — Schematic of instrument. Key-DCSP - dichroic short pass, DCLP – dichroic long pass. (PDF) [file pone.0043460.s004.pdf]
