# Supplementary material for: Multiphoton Multispectral Fluorescence Lifetime Tomography for the Evaluation of Basal Cell Carcinomas
Source: PLoS One. 2012 Sep 11;7(9):e43460. doi: 10.1371/journal.pone.0043460 (PMC3439453; doi:10.1371/journal.pone.0043460)

Figure S5 - Bar chart showing sample median ROI/cell mean fluorescence lifetime for BCCs, *in vivo* normal and *ex vivo* normal skin for the four spectral detection channels. Error bars indicate value of 25th and 75th percentile.

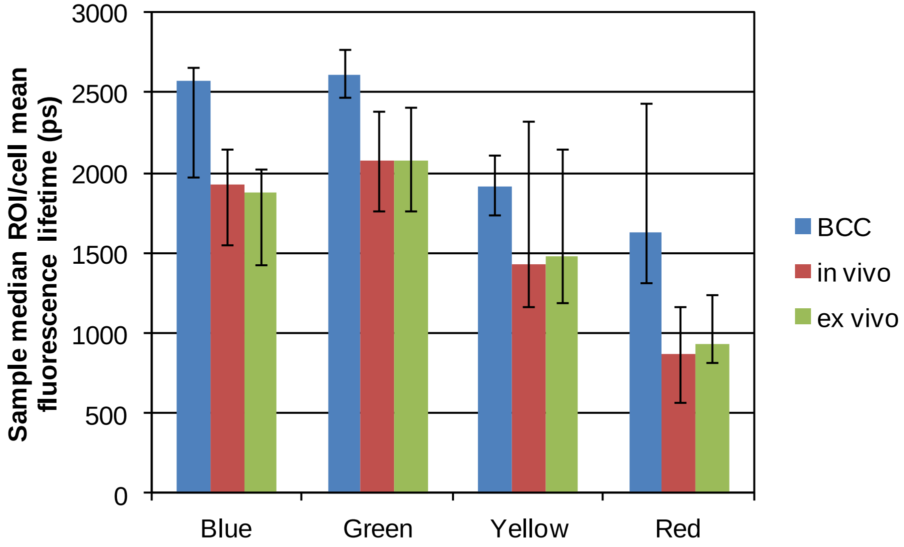

Supplement: Figure S5 — Bar chart showing sample median ROI/cell mean fluorescence lifetime for BCCs, in vivo normal and ex vivo normal skin for the four spectral detection channels. Error bars indicate value of 25th and 75th percentile. (PDF) [file pone.0043460.s005.pdf]
