# Supplementary material for: Multiphoton Multispectral Fluorescence Lifetime Tomography for the Evaluation of Basal Cell Carcinomas
Source: PLoS One. 2012 Sep 11;7(9):e43460. doi: 10.1371/journal.pone.0043460 (PMC3439453; doi:10.1371/journal.pone.0043460)
Supplement: Table S1 — Summary of all spectroscopic parameters calculated for each ROI/cell. (PDF) [file pone.0043460.s006.pdf]

Table S1 – Summary of all spectroscopic parameters calculated for each ROI/cell

Index	Channel	Diagnosis	Percentile 25	50	75
τ_{mean} (ps)	Blue	BCC	1759	2419	2773
		Normal	1282	1797	2175
	Green	BCC	2412	2624	2819
		Normal	1656	2189	2709
	Yellow	BCC	1582	1908	2200
		Normal	888	1380	1974
	Red	BCC	1240	1448	2013
		Normal	709	1036	1428
f_1	Blue	BCC	0.268	0.312	0.428
		Normal	0.299	0.397	0.589
	Green	BCC	0.326	0.349	0.380
		Normal	0.307	0.342	0.415
	Yellow	BCC	0.378	0.406	0.448
		Normal	0.374	0.455	0.617
	Red	BCC	0.490	0.532	0.585
		Normal	0.488	0.564	0.652
τ_1 (ps)	Blue	BCC	56	115	302
		Normal	34	47	71
	Green	BCC	325	418	470
		Normal	67	178	397
	Yellow	BCC	187	293	368
		Normal	53	94	232
	Red	BCC	200	263	388
		Normal	80	130	208
τ_2 (ps)	Blue	BCC	3053	3379	3766
		Normal	2800	2986	3205
	Green	BCC	3512	3857	4122
		Normal	2812	3141	3786
	Yellow	BCC	2630	3058	3476
		Normal	2217	2475	2985
	Red	BCC	2301	2917	3790
		Normal	1803	2211	2634
Spectral Contribution	Blue	BCC	0.2533	0.2740	0.3149
		Normal	0.2263	0.2527	0.2964
	Green	BCC	0.5269	0.5633	0.5923
		Normal	0.5301	0.5653	0.6633
	Yellow	BCC	0.1199	0.1601	0.1994
		Normal	0.0314	0.1828	0.2185
	Red	BCC	0.0002	0.0006	0.0036
		Normal	0.0001	0.0005	0.0011

**Total Photons per ROI**	Blue	BCC	5722	11739	22650
		Normal	2363	5540	11220
	Green	BCC	32062	66009	120605
		Normal	17529	31816	58006
	Yellow	BCC	15051	28605	54824
		Normal	10994	20143	37669
	Red	BCC	743	3822	24041
		Normal	4386	8837	15841
