# Supplementary material for: Multiphoton Multispectral Fluorescence Lifetime Tomography for the Evaluation of Basal Cell Carcinomas
Source: PLoS One. 2012 Sep 11;7(9):e43460. doi: 10.1371/journal.pone.0043460 (PMC3439453; doi:10.1371/journal.pone.0043460)
Supplement: Table S2 — The AUC for all calculated spectroscopic (a) and cellular morphology parameters (b). (PDF) [file pone.0043460.s007.pdf]

Table S2 - The AUC for all calculated spectroscopic (a) and cellular morphology parameters (b).

Parameter	Index	Channel	Cohen's d	AUC
**Fluorescence Intensity**	Spectral Contribution	Blue	0.45	0.63
	Spectral Contribution	Red	0.73	0.61
	Fluorescence Coefficient of Variation	Yellow	0.17	0.59
	Spectral Contribution	Green	0.48	0.58
	Fluorescence Coefficient of Variation	Blue	0.17	0.53
	Spectral Contribution	Yellow	0.03	0.52
	Fluorescence Coefficient of Variation	Red	0.20	0.52
	Fluorescence Coefficient of Variation	Green	0.06	0.51
**Fluorescence Lifetime**	τ_1	Red	1.44	0.82
	τ_1	Blue	1.22	0.80
	τ_1	Yellow	1.00	0.77
	τ_2	Red	0.20	0.74
	τ_2	Green	0.87	0.73
	τ_2	Blue	0.38	0.73
	τ_1	Green	0.83	0.72
	τ_2	Yellow	0.53	0.71
	f_1	Blue	0.40	0.63
	f_1	Yellow	0.57	0.62
	f_1	Red	0.34	0.59
	f_1	Green	0.29	0.52

(a)

Parameter	Index	Adjacency Calculation Method	Cohen's d	AUC
**Cellular morphology**	Number of Neighbours	Delaunay	0.14	0.66
	Number of Neighbours	Gabriel	0.12	0.64
	Number of Neighbours	SOI	0.17	0.62
	Cell Confluency	Gabriel	0.02	0.60
	Cell Confluency	SOI	0.02	0.60
	Cell Confluency	Delaunay	0.29	0.60
	Solidity		0.27	0.58
	Area		0.00	0.58
	Cell Density	Delaunay	0.29	0.57

Cell Density	SOI	0.02	0.57
Distance to Neighbours	SOI	0.14	0.57
Distance to Neighbours	Delaunay	0.11	0.56
Cell Density	Gabriel	0.02	0.56
Distance to Neighbours	Gabriel	0.11	0.56
Distance to Neighbours	SOI	0.13	0.56
Distance to Neighbours	Delaunay	0.04	0.55
Distance to Neighbours	Gabriel	0.02	0.54
Orientation SD	Gabriel	0.12	0.53
Orientation SD	Delaunay	0.14	0.53
Orientation SD	SOI	0.13	0.53
Flattening Factor	SOI	0.18	0.53
Flattening Factor	Gabriel	0.17	0.52
Gyration Radius		0.16	0.52
Flattening Factor	Delaunay	0.16	0.52
Shape Factor		0.05	0.52

(b)
