# Supplementary material for: Multiphoton Multispectral Fluorescence Lifetime Tomography for the Evaluation of Basal Cell Carcinomas
Source: PLoS One. 2012 Sep 11;7(9):e43460. doi: 10.1371/journal.pone.0043460 (PMC3439453; doi:10.1371/journal.pone.0043460)
Supplement: Text S1 — Supporting Materials and Methods. (PDF) [file pone.0043460.s008.pdf]

Text S1 - Supporting Materials and Methods

Instrument

The DermaInspect instrument incorporates a modelocked 80 MHz Titanium:Sapphire laser (MaiTai, Spectra Physics®, CA, USA) equipped with a prism-based chirp compensation unit (DeepSee™ module, Spectra Physics®). The laser has a tuning range between 710-920 nm and the excitation power was attenuated to a maximum of 50 mW at the sample (measured at 800 nm). The excitation beam passes through two galvoscanners, allowing scanning in the x, y plane, before passing through a 40x/1.4NA oil immersion objective. The focusing optics allow a working depth of up to 200 µm with a lateral resolution of <1 µm and axial resolution of <2 µm.

Ex vivo specimens in an inverted glass bottomed petri dish were coupled to the DermaInspect® magnetically using a metallic coupling ring attached to the dish with an adhesive tape. Immersion oil (Immersol™ 518F, Carl Zeiss Ltd, Germany) was placed between the glass and the objective. For *in vivo* imaging, a glass coverslip was attached to the metallic ring and then attached to the patient using an adhesive tape. A drop of water was placed between the patient's skin and the coverslip, and immersion oil was again placed between the glass and the objective.

The multispectral FLIM detector has been described previously [1,2]. Minor modifications were made to the system during the study to broaden the longest wavelength channel's detection bandwidth from 620-640 to 620-655 nm and to improve the spectral separation between the 360-425 nm and 425-515 nm channels. To achieve this, the 640 nm DCSP (Chroma Technology Corp., Vermont, USA; position 1 figure S4) was replaced with a 670 nm DCSP, the BG39 filter (position 2) was replaced with a 680 nm short pass filter and a short pass 440 nm filter was inserted in position 3. The change in filters does not affect the spectral contribution measurements due to the daily calibration of the spectral response of the system.

Cellular morphology quantifiers

The cellular morphology quantifiers were based on cell ROI geometry and are defined in the table below. They include individual ROI measurements such as Area, Perimeter, Gyration Radius and the Shape Factor. A standard MATLAB morphometry routine "regionprops" was applied to obtain several more geometric features such as Solidity and Major and Minor Axis Lengths. The dimensional geometric parameters (e.g. area and distance measured in microns) were calculated taking into account image magnification.

Parameter	Description
Perimeter	Perimeter of ROI's boundary (µm)
Major Axis Length	The length of major axis of the effective ellipse (µm)
Minor Axis Length	The length of minor axis of the effective ellipse (µm)
Gyration Radius	A linear measure of distance of a point in the ROI from the centre, an indirect measure of effective linear size (µm)
Area	Area of ROI (μm^2)
Shape Factor	$Perimeter^2/(4\pi Area)$. A measure of how circular an object is (equals 1 for a circle and >1 for any other figure)
Solidity	Ratio of cell area/area of convex hull. A measure of negative curvature. Equals maximum 1 for shape with no concave parts e.g. a circle or square
Flattening Factor	Ratio of minor to major axis. A measure of flatness.
Cell Density	The effective number of ROIs per μm^2
Cell Confluency	Relative area occupied in the image by a ROI and its neighbouring ROIs. Indirect measure of the area of cytoplasm present and therefore scales inversely with the amount of extracellular matrix present.
Orientation SD	Standard deviation of the angle of the major axis of a ROI and its neighbouring ROIs (degrees)
Number of Neighbours	The number of neighbouring ROIs
Distance to Neighbours	The average distance to a neighbouring ROI (µm)

Another group of morphology features was designed to study properties of groups of neighbouring cells. These methods first define local neighbourhood relations via adjacency graphs in the image. The Delaunay triangulation, Gabriel and Sphere of Influence (SOI) graphs [3;P245] were constructed for each field of view, typically encompassing several dozens of cells (ROIs) in the image. Every cell was characterized (for every adjacency graph) by its number of neighbours n_{nb} and average distance to the neighbour d_{nb}. Local cell density and the cell confluency were estimated via the area of the effective influence circle $A_{inf} = \pi {d_{nb}}^2$:

$$Cell\ Density = \frac{1 + \sum_{k=1}^{n_{nb}} \frac{1}{n_{nb(k)}}}{A_{inf}}$$

$$Cell\ Confluency = \frac{\frac{1}{n_{nb}+1}\left(\sum_{k=1}^{n_{nb}} Area_{(k)} + Area_{cell}\right)}{A_{inf}}$$

where the (*k*)-index summation is extended over a cell's nearest neighbours. Other local statistics were calculated per cell with the help of the statistical sample including that cell and its nearest neighbours.

Fluorescence intensity/spectral contribution

For the purpose of intensity and spectral analysis, the total intensity *I* was calculated by integrating over the fluorescence decay. Quantifiers included the total number of photons per ROI and the coefficient of variation (*CV*) (defined as the ratio of standard deviation of ROI pixels' intensities to their mean). In order to exclude the contribution of the Poisson pixel noise to *CV* values, the corrected formula $CV = \left[Var_{ROI}(I)/E_{ROI}(I)^2 - 1/E_{ROI}(I)\right]^{1/2}$ was applied, where Var_{ROI} and E_{ROI} are the estimates of the variance and expectation (average) of the intensity respectively.

The spectral contribution of each channel was determined by calculating the proportion of the total fluorescence signal in that channel [4]. The total fluorescence signal was first corrected for the PMT background and after-pulsing effects, and then calibrated against the signal acquired daily from a reference fluorophore with a known fluorescence emission spectrum (blue fluorescent slide available from Chroma Technology Corp., Vermont, USA).

Fluorescence decay analysis

Two fluorescence decay models were used. A single exponential decay model was used to fit each pixel of the FLIM images for the visual architectural assessment. This was chosen because it serves as a convenient parameter to visually assess fluorescence lifetime changes over an entire image and because insufficient photons are collected from each pixel to fit a more complex fluorescence decay model. Decay data was smoothed spatially prior to fitting (5x5 kernel for 256x256 images; 3x3 kernel for 128x128 images) and the resulting FLIM map merged with the fluorescence intensity image. Where necessary, images were resized using bilinear interpolation.

Our method of ROI fluorescence decay analysis has been presented elsewhere [4]. Briefly, the ROI decay was calculated by summing photons within each ROI at each time delay. The fitting model included the measured background count rate and the measured after-pulsing

probability for each detector. The analysis in this paper used the 'data-weighted' definition of the fitting error function χ^2 as it was found to be less biased when fitting a double exponential decay model.

$$\chi^2 = \sum_{k=1}^{N_{bins}} \frac{[I_{\text{model}}(t_k) - I_{\text{measured}}(t_k)]^2}{I_{\text{measured}}(t_k)}$$

Here, t_k is the time delay of the kth bin, I_{model} is the decay model convolved with the measured Instrument Response Function and I_{measured} is the recorded fluorescence signal at each time delay. The fluorescence lifetime parameters comprise the fitted bi-exponential lifetimes τ_1, τ_2, and the f_1 parameter ($0< f_1<1$), which is the fraction of photons originating from the short (τ_1) decay. Mean fluorescence lifetimes were calculated as $\tau_{mean}= f_1 \tau_1+(1–f_1) \tau_2$.

The application of ROIs to define cells allowed a threshold of 1000 photons to be reached for most ROIs, a level we felt sufficient to reliably fit a double exponential decay model for the decay analysis. A double exponential decay model was chosen as it provides a reasonable fit to the data and allows some information to be gleaned regarding the complex nature of the underlying fluorescence decay, which is considerably more complex than this. For example, free NADH in solution is known to have a double exponential decay and the fluorescence lifetimes of both components can change when they bind to proteins. Furthermore, there are a large number of potential protein binding partners and therefore more than 4 decay components are required for intracellular NADH alone [5]. Similarly, melanin is known to have at least 3 fluorescence decay components [6]. Given the limited number of detected photons available, we therefore chose to fit only two decay components to the fluorescence signal from a cellular region of interest in all spectral channels. Therefore, the short and long fluorescence decay components (τ_1 and τ_2) serve to summarize the changes occurring in a complex biological system exhibiting a multi-exponential decay. The blue, green and yellow channels had 93-100% of ROIs reaching the 1000 photon threshold for fitting in both diagnostic groups. 68.0% ROIs from BCC vs 93.9% ROIs from normal skin were sufficiently bright in the red channel for lifetimes to be calculated (see total photon count per ROI statistics in Table S1).

In order to quantify the accuracy of our fitting algorithm, we simulated a representative fluorescence decay with the following parameters: τ_1 = 400 ps, τ_2 = 3000 ps, f_1 = 0.35 and total number of photons = 10,000. We then convolved the simulated decay with a typical experimentally recorded IRF, added Poisson noise to the simulated fluorescence decay, and repeated this process to generate a total of 1000 simulated noisy decay curves. We then fitted these simulated decays using the same fitting code that was used for analysing the experimental data and calculated the mean and standard deviation of each of the parameters fitted. The standard deviation and bias (mean fitted value minus simulated value) of all fit parameters was found to be less than 5% of the simulated value. The standard deviation and bias of the mean fluorescence lifetime calculated from the double exponential fit were also found to be less than 5% of the simulated value.

Statistics

The Wilcoxon rank sum nonparametric test [7;P119-28] was applied to study the difference in distribution between BCC and normal groups using MATLAB.

The discriminative ability of the ROI measurements were assessed using 2 parameters: Receiver Operator Characteristic Area Under the Curve (AUC,[8;P158]) and the Cohen's d statistic [9;P262]. See table 2, supplementary material 5.

In total, 4259 BCC and 6203 normal cell ROIs were identified manually. The number of ROIs used for the fluorescence lifetime parameters was lower, as not all ROIs reached the threshold of 1000 photons (see fluorescence decay analysis section above).

Principal Component Analysis/Linear discriminant analysis discrimination

For the manually segmented ROI data, all spectroscopic and cellular morphology parameters were included in a correlation-matrix based principal component analysis [10;P24]. The first 4 principal components were selected to reduce the dimensionality of the data. Linear discriminant analysis was then applied to the selected principal components for each ROI using a leave one out approach. The fraction of ROIs classified as a BCC was then calculated for each patient. For the automatically segmented data, only the spectroscopic parameters were included in the analysis.

Unsegmented images were analyzed by first spatially integrating the fluorescence decay profiles over each field of view, fitting the resulting decays and then calculating the mean spectroscopic parameters for each patient. These mean parameters were then included in a principal component analysis and the first 4 components were selected. Linear discriminant analysis was then applied to the selected principal components using a leave one out approach and the sensitivity and specificity were then calculated.

References

1. Talbot CB, Patalay R, Munro I, Breunig HG, König K, et al. A multispectral FLIM tomograph for in-vivo imaging of skin cancer. In: Periasamy A, Konig K, So PTC, editors. Proc. SPIE; 2011; San Francisco, California, USA. Proc. SPIE. pp. 79032B-79039.
2. Talbot CB, Patalay R, Munro I, Warren S, Ratto F, et al. (2011) Application of ultrafast gold luminescence to measuring the instrument response function for multispectral multiphoton fluorescence lifetime imaging. Optics Express 19: 13848-13861.
3. Bivand RS, Pebesma EJ, Gomez-Rubio V (2008) Applied Spatial Data Analysis with R: Springer Science & Business Media.
4. Patalay R, Talbot C, Alexandrov Y, Munro I, Neil MAA, et al. (2011) Quantification of cellular autofluorescence of human skin using multiphoton tomography and fluorescence lifetime imaging in two spectral detection channels. Biomed Opt Express 2: 3295-3308.

5. Vishwasrao HD, Heikal AA, Kasischke KA, Webb WW (2005) Conformational dependence of intracellular NADH on metabolic state revealed by associated fluorescence anisotropy. J Biol Chem 280: 25119-25126.
6. Teuchner K, Freyer W, Leupold D, Volkmer A, Birch DJ, et al. (1999) Femtosecond two-photon excited fluorescence of melanin. Photochem Photobiol 70: 146-151.
7. Lupton R (1993) Statistics in theory and practice: Princeton University Press.
8. Mathews (2010) Sample Size Calculations: Practical Methods for Engineers and Scientists: Mathews Malnar and Bailey Inc.
9. Gravetter FJ, Wallnau LB (2009) Statistics for the Behavioral Sciences: Wadsworth.
10. Joliffe IT (2002) Principal Component Analysis. New York: Springer-Verlag.
